# The oncolytic bacteria-mediated delivery system of CCDC25 nucleic acid drug inhibits neutrophil extracellular traps induced tumor metastasis

**DOI:** 10.1186/s12951-024-02335-5

**Published:** 2024-02-19

**Authors:** Li-na Liu, Chen Chen, Wen-jie Xin, Qiang Li, Chao Han, Zi-chun Hua

**Affiliations:** 1 The State Key Laboratory of Pharmaceutical Biotechnology, School of Life Sciences, Nanjing University, 163 Xianlin Avenue, Nanjing, 210023 Jiangsu China; 2 Changzhou High-Tech Research Institute of Nanjing University and Jiangsu, Changzhou, China; 3 TargetPharma Laboratories Inc., Changzhou, 213164 Jiangsu China

**Keywords:** VNP20009, NETs, CCDC25, Neutrophils, Metastasis, Nucleic acid delivery

## Abstract

**Background:**

Neutrophil extracellular traps (NETs), antibacterial weapons of neutrophils (NEs), have been found to play a crucial role in cancer metastasis in recent years. More and more cancer research is focusing on anti-NETs. However, almost all anti-NETs treatments have limitations such as large side effects and limited efficacy. Therefore, exploring new anti-NETs therapeutic strategies is a long-term goal.

**Results:**

The transmembrane protein coiled-coil domain containing 25 (CCDC25) on tumor cell membranes can bind NETs-DNA with high specificity and affinity, enabling tumor cells to sense NETs and thus promote distant metastasis. We transformed shCCDC25 into VNP20009 (VNP), an oncolytic bacterium, to generate VNP-shCCDC25 and performed preclinical evaluation of the inhibitory effect of shCCDC25 on cancer metastasis in B16F10 lung metastasis and 4T1 orthotopic lung metastasis models. VNP-shCCDC25 effectively blocked the downstream prometastatic signaling pathway of *CCDC25* at tumor sites and reduced the formation of NETs while recruiting more neutrophils and macrophages to the tumor core, ultimately leading to excellent metastasis inhibition in the two lung metastasis models.

**Conclusion:**

This study is a pioneer in focusing on the effect of anti-NET treatment on *CCDC25*. shCCDC25 is effectively delivered to tumor sites via the help of oncolytic bacteria and has broad application in the inhibition of cancer metastasis via anti-NETs.

**Graphical Abstract:**

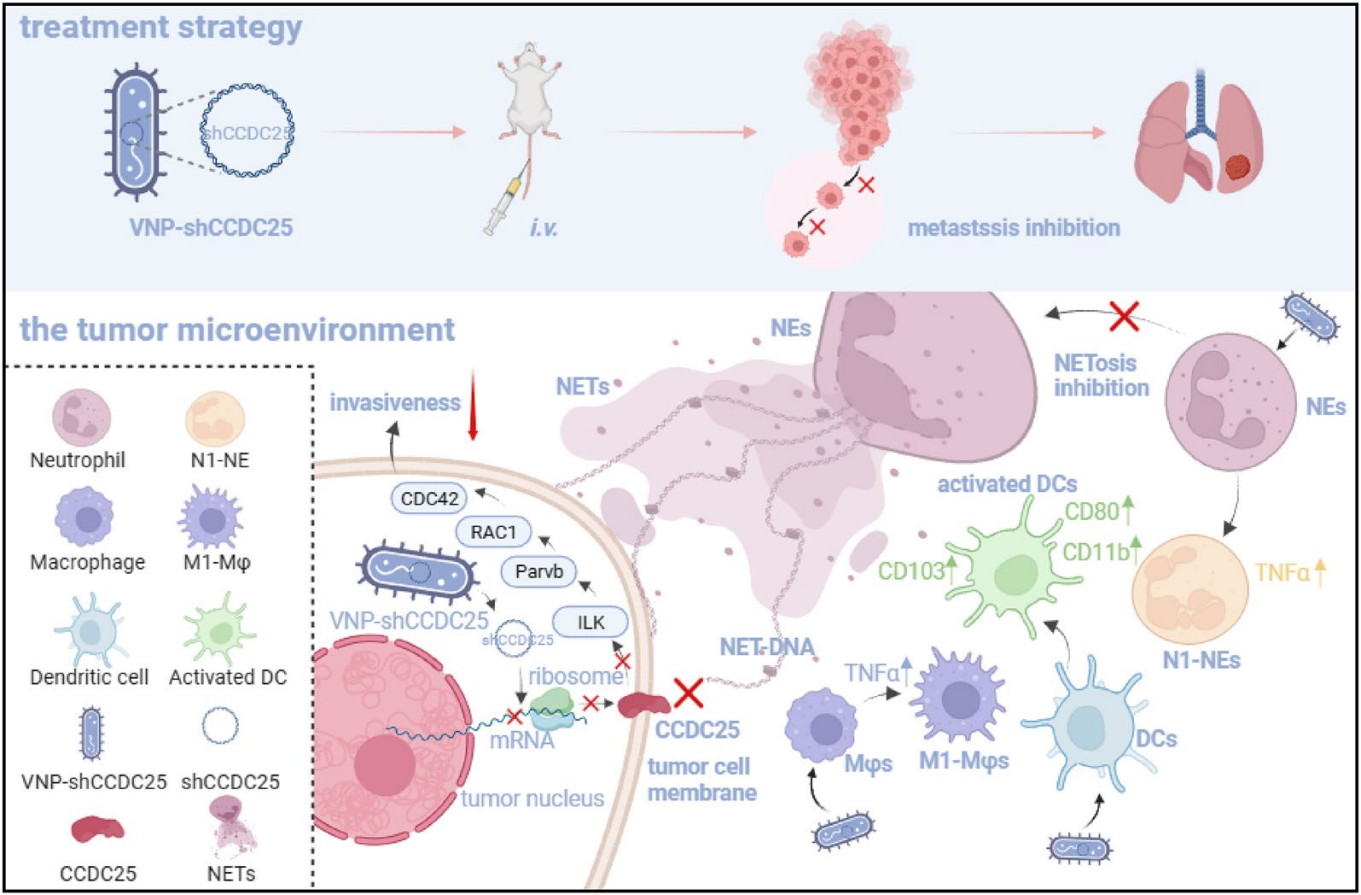

**Supplementary Information:**

The online version contains supplementary material available at 10.1186/s12951-024-02335-5.

## Introduction

A key feature of neutrophils (NEs) as a host defense against pathogens is their ability to extrude a specialized structure called neutrophil extracellular traps (NETs) into the surrounding environment [1]. NETs are composed of chromatin DNA filaments encased in granule proteins that can serve as antibacterial weapons for NEs [2]. Specifically, peptidyl arginine deiminase 4 (PAD4) is localized NE nuclei and modifies histones by converting arginine to citrulline, leading to chromatin decondensation; after nuclear rupture, citrullinated histones are released along with nuclear DNA, and the released DNA is further modified by granular neutrophil elastase (NE), myeloperoxidase (MPO), and cytoplasmic proteins, eventually leading to the formation of NETs. In recent years, reports of tumor-associated neutrophils (TANs) have led to a new understanding of the functional diversity of NEs [3]. As pathogens, tumor cells recruit NEs to the tumor microenvironment (TME), after which NEs are activated and release NETs, which in turn promote tumor growth and metastasis [4]. Excessive formation or impaired degradation of NETs is a key event in cancer metastasis. Therefore, reducing the formation or enhancing the degradation of NETs at the tumor site is a cancer treatment strategy [3].

In recent years, an increasing number of cancer studies have focused on NETs, but ongoing clinical trials have not yet clarified the optimal mechanism of action of NETs [5, 6]. Most related studies have focused on NEs, but NEs are short-lived and can be recruited rapidly; moreover, primary human NEs cannot be transfected, and it is difficult to specifically inhibit NET production from TANs in vivo [3]. Surprisingly, one study revealed that a large number of NETs were present at the site of tumor metastasis in breast cancer patients, and further studies revealed that NETs mediate the distant metastasis of breast cancer cells via the receptor coiled-coil domain containing 25 (CCDC25), which binds to NETs-DNA [7]. CCDC25 is a transmembrane protein located on the cell membranes of cancer cells that can bind to NETs-DNA with high specificity and affinity, enabling cancer cells to sense NETs and thus promote distant metastasis [7]. Therefore, targeted inhibition of CCDC25 at tumor sites is expected to be a new and efficient cancer treatment strategy. CCDC25 can be directly targeted and blocked by antibodies, but these antibodies are costly and require multiple doses. In comparison, nucleic acid drugs have the advantages of high efficiency and low cost. However, effective delivery of nucleic acid drugs is a major challenge due to their negative charge, hydrophilicity, inability to directly penetrate cell membranes, and susceptibility to enzymatic degradation [8].

VNP, obtained by knocking out virulence genes in *Salmonella typhimurium*, has the advantages of good therapeutic efficacy and few side effects. In addition, VNP can destroy tumor tissue by competing for nutrients, secreting toxins, and triggering host immune responses, and it has been shown to be safe in phase I clinical trials [9–11]. As a typical oncolytic bacterium, VNP can encapsulate nucleic acid drugs, target tumors, and infest tumor cells, and upon intracellular lysis, release the nucleic acid drugs carried within the VNP [12]. In this study, we aimed to deliver an encapsulated nucleic acid drug to the tumor site via delivery of VNP to ultimately achieve the goal of targeting the *CCDC25* gene and thus inhibiting tumor metastasis.

## Materials and methods

### Bacterial strains and plasmid

Lipid A modified (*msbB*^*−*^), auxotrophic (*purI*^*−*^) *Salmonella typhimurium* VNP20009 (VNP), and J23100 initiate VNP expressing red fluorescent protein (RFP) (VNP-RFP) were stored in our lab and cultured in modified Luria–Bertani (LB) media, at 37 ℃. B16F10 cells were subjected to shRNA against *CCDC25* transfection, in order to determine the interference efficiency and measured by qPCR. The pRNA U6.1 vector containing the 21-mer shRNA sense sequence 5’-GCTGTGGATCTTGGGATATCC-3’ can effectively silence *CCDC25*. The pRNA U6.1 containing the shRNA sequence was electroporated into VNP by Gene Pulser Xcell (Bio-Rad).

### Cell lines

The B16F10 cells (mouse melanoma cell) and the 4T1 cells (mouse breast cancer cell) were stored in our lab. They were both cultured in RPMI-1640 medium (BBI, China) containing 10% fetal bovine serum (FBS) (HyClone, USA). The RAW 264.7 cells (mouse macrophage cell) were stored in our lab and cultured in Dulbecco's modified eagle medium (DMEM) (BBI, China) containing 15% FBS (Gibco, USA).

### Animals and animal models

Female C57BL/6 and BALB/c, 6–8 weeks, were purchased from Huachuang Sino Company (Nanjing, China) and housed under constant pathogen-free conditions. 2 × 10^5^ B16F10 cells re-suspended in 100 *μL* of PBS were intravenous (*i.v.*) into the C57BL/6 mice to establish a lung metastases model. 1 × 10^5^ 4T1 cells re-suspended in 20 *μL* of PBS were injected in the fourth mammary fat pads on one flank of the BALB/c mice to establish orthotopic model of breast cancer.

### Animal experiments

Three days after implantation, PBS, 1 × 10^6^ CFU VNP-NC and 1 × 10^6^ CFU VNP-shCCDC25 were injected respectably by intravenous injection into tumor-bearing C57BL/6 mice. Then 5 days after administration, the mice were executed and collected peripheral blood, heart, liver, spleen, lung, kidney, and tumor-draining lymph nodes (TdLNs) for further analysis. Sixteen days after implantation, breast carcinoma in situ of the BALB/c mice was removed through surgery. Two days after surgery, PBS, 1 × 10^6^ CFU VNP-NC, 1 × 10^6^ CFU VNP-shCCDC25 was injected respectably by intravenous injection into the BALB/c mice. Then ten days after administration, the mice were executed and the collected peripheral blood, heart, liver, spleen, lung, kidney, and TdLNs were for further analysis. The body weight was measured daily. To comply with ethical requirements, the animal experiments, some of the control groups (PBS group, VNP-NC group) were combined with our previous work, which has been published online under the name of “Neutrophil-Mediated Tumor-Targeting Delivery System of Oncolytic Bacteria Combined with ICB for Melanoma Lung Metastasis Therapy” [13]. The tumor inhibition rates were calculated as follows: tumor inhibition rate = (1-average tumor foci numbers of the treatment group/average tumor foci numbers of control group) × 100.

### Bacterial titer of tumor metastases

After administration, pulmonary metastasis was collected and lysed using 1% Triton X-100 at 1 h at 4 ℃. The supernatants were planted on LB agar after being diluted in PBS, and the bacterial numbers were calculated.

### Serological test, routine blood test and H&E staining

Serum samples were collected to detect aspartate aminotransferase (AST), alanine aminotransferase (ALT), creatinine (Scr), and blood urea nitrogen (BUN). Whole blood samples were collected for routine blood test. Mouse organs and tumors were collected for H&E staining. These experiments were completed by the Wuhan Servicebio technology company (Wuhan, China).

### Immunohistochemistry

Whole mouse lungs were fixed in 4% paraformaldehyde solution for 72 h. The fixed lungs were embedded in paraffin and cut to a thickness of 5 μm. Sections were placed on glass slides, dewaxed, and hydrated, and subjected to standard H&E staining. They were incubated with anti-MPO, developed by DAB (Servicebio, Wuhan, China) for 5 min, and finally observed under a microscope.

### Bacterial infestation of B16F10 cells assay

B16F10 cells were seeded at a density of 2 × 10^5^ per well in 12-well plates. VNP-NC, VNP-shCCDC25 or not was added to each well and incubated for 16 h (MOI = 100:1). Cells were lysed using 1% Triton X-100 at 1 h at 4 ℃. The supernatants were planted on LB agar after being diluted in PBS, and the bacterial numbers were calculated.

### Bacterial stimulation of RAW264.7 cells assay

RAW264.7 cells were seeded at a density of 4 × 10^5^ per well in 6-well plates. VNP-NC, VNP-shCCDC25 (MOI = 100:1) or not was added to each well and incubation for 2 h. Cells were collected for further experiments.

### qPCR assay

Total RNA was isolated with Trizol reagent (Invitrogen). cDNA was generalized using ReverTra Ace® qPCR RT Kit (Toyobo). qPCR was done with primers (detailed sequence information is provided in Additional file 1: Table S1 to determine the mRNA expression level of the target gene. qPCR was performed on StepOne Real-Time PCR System (Applied Biosystems, USA) with AceQ® qPCR SYBR® Green Master Mix (V azyme China). Data were analyzed by StepOne Software 2.1 (Applied Biosystems, USA) according to the manufacturer's specifications. 18S rRNA was used as a control.

### Apoptosis detection by Annexin V⁄PI staining

B16F10 cells were seeded at a density of 2 × 10^5^ per well in 24-well plates and incubated with different bacteria or bacteria-stimulated RAW264.7 cell medium for 16 h. The apoptosis levels of B16F10 cells were determined using a kit developed by our lab. 1 *µL* Annexin V-APC (1 mg*/ml*) and 1 *µL* propidium-PE (PI, 1 mg*/ml*) were incubated with cells in binding buffer for 30 min at 4 ℃. The stained cells were analyzed using FACS (NovoCyte Flow Cytometer (ACEA@)). The results were analyzed using FlowJo VX software.

### Flow cytometry

5 days after administration, tumor-bearing mouse tissues were collected. Spleen and TdLNs were homogenized with 1 ml PBS to obtain single-cell suspensions. Peripheral blood lymphocytes were obtained from peripheral blood. Tumors on the lung were shredded and then digested with mixed medium (1 mg*/ml* Collagenase I, 1 mg*/ml* Collagenase IV, 200 µg*/ml* DNase I) at 37 ℃ for 40 min. All tissues were lysed with red blood cell lysis buffer (Beyotime, Nanjing), and then the cell suspensions were passed through a 200-mesh filter. The single-cell suspensions were incubated in 1% BSA for 15 min at 4 ℃ and stained with the following antibodies for 30 min at 4 ℃ (detailed antibody information is provided in Additional file 1: Table S2). The stained cells were analyzed using flow cytometer (BD@ FACS Canto II systems). The results were analyzed using FlowJo VX software.

### Statistical analysis

Results are expressed as the mean ± SD as specified. Mean differences were compared using t-test or one-way ANOVA. A value of *P* < 0.05 was regarded as statistically significant. Data were analyzed with GraphPad Prism 8.3 software. (**** *P* < 0.0001, *** *P* < 0.001, ** *P* < 0.01, * *P* < 0.05.)

## Results

### Construction and characterization of VNP-shCCDC25

**Fig. 1:**
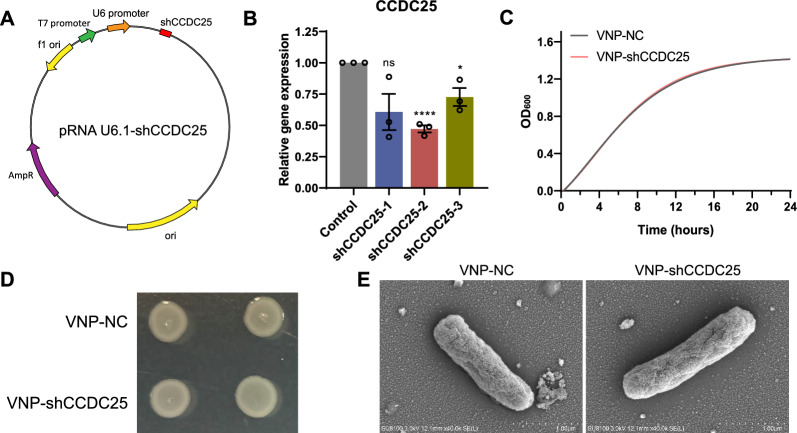
Construction and characterization of VNP-shCCDC25. **A** Schematic diagram of pRNA U6.1- shCCDC25. **B** The expression of *CCDC25* in B16F10 cells after transfection with pRNA U6.1-shCCDC25 was analyzed via qPCR. **C** Bacteria growth curves of VNP-NC and VNP-shCCDC25 (*n* = 6). **D** Colonial morphology of VNP-NC and VNP-shCCDC25. **E** Scanning electron microscopes (SEM) of VNP-NC and VNP-shCCDC25. Data are shown as the mean ± SD. **** p < 0.0001, *** p < 0.001, ** p < 0.01, * p < 0.05, ns: no significance

Considering that *CCDC25* is an attractive target, three eukaryotic interference plasmids were constructed to silence *CCDC25* (Fig. 1A) and were transfected into B16F10 cells separately. qPCR results demonstrated that pRNA U6.1-shCCDC25-2 (shCCDC25) could effectively knock down *CCDC25* by more than 50% (Fig. 1B). Therefore, we selected this interference plasmid for further studies. To achieve effective silencing in vivo, the drug-loaded bacterium VNP-shCCDC25 was generated by transforming shCCDC25 into VNP, and U6.1-NC pRNA was transformed into VNP (*i.e.*, VNP-NC) to serve as a control. To exclude the interference plasmid from affecting the normal growth of the bacteria, the growth curve was monitored (Fig. 1C), and the results revealed that the growth status of VNP-shCCDC25 was consistent with that of VNP-NC. In addition, the morphology of individual VNP-shCCDC25 colonies was observed (Fig. 1D). The morphology of individual VNP-shCCDC25 cells was observed by scanning electron microscopy (SEM) (Fig. 1E). The results indicated that the normal growth of bacteria was not affected after the transformation of the interfering plasmid shCCDC25 into VNP.

### VNP-shCCDC25 stimulates neutrophil polarization to N1 and macrophage polarization to M1, infects cells and knocks down *CCDC25 *in vitro

**Fig. 2:**
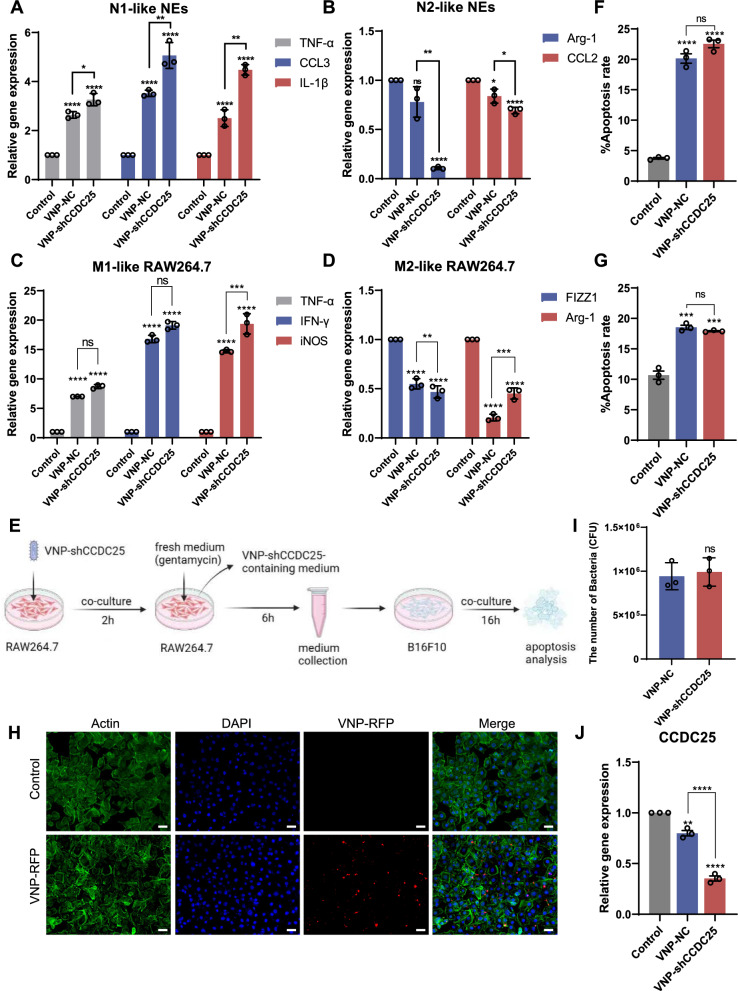
VNP-shCCDC25 stimulates NEs and macrophage activation and infect cells in vitro. **A** The expression of N1 polarization markers of NEs after VNP-shCCDC25 treatment was analyzed via qPCR. **B** The expression of N2 polarization markers of NEs after VNP-shCCDC25 treatment was analyzed via qPCR. **C** The expression of M1 polarization markers of RAW264.7 cells after VNP-shCCDC25 treatment was analyzed via qPCR. **D** The expression of M2 polarization markers of RAW264.7 cells after VNP-shCCDC25 treatment was analyzed via qPCR. **E** Schematic diagram of RAW264.7 cell medium stimulated by VNP-shCCDC25 promotes apoptosis of B16F10 cells. After VNP-shCCDC25 was co-cultured with RAW264.7 cells for 2 h, the medium was changed to fresh medium containing gentamycin, and the incubation was continued for 6 h. After 6 h, the RAW264.7 cell medium was collected and co-incubated with B16F10 cells for 16 h. Finally, B16F10 cells were collected for the apoptosis assay. **F** The apoptosis levels of B16F10 cells after incubated for 16 h with RAW264.7 cell medium, which was stimulated with VNP-NC, VNP-shCCDC25, or not (*n* = 3). **G** The apoptosis levels of B16F10 cells after incubated with VNP-NC, VNP-shCCDC25, or not for 16 h (*n* = 3). **H** The fluorescent pictures of B16F10 cells incubated with VNP-RFP (MOI = 100:1) or not; actin (green), DAPI (blue), VNP-RFP (red). Scale bars: 25 μm. **I** The titer of bacterium colonized in the B16F10 cells after co-incubated with VNP-NC or VNP-shCCDC25 (*n* = 3) for 16 h. **J** The expression of *CCDC25* in B16F10 cells after incubated with VNP-shCCDC25 was analyzed via qPCR. Data are shown as the mean ± SD. **** p < 0.0001, *** p < 0.001, ** p < 0.01, * p < 0.05, ns: no significance

Many studies have shown that the injection of specific bacteria can suppress tumors by stimulating inflammation and triggering antitumor immune responses, which involves immune cells including NEs and macrophages (Mφs) [14]. It has been proposed that in the TME, TANs exhibit an N1 phenotype, which is an antitumor phenotype, and an N2 phenotype, which is a protumor phenotype [15]. It has been shown that *TNF-α*, *CCL3*, and *IL-1β* are markers for N1-NEs [15], and *Arg-1* and *CCL2* are markers for N2-NEs [16]. Based on this, we stimulated NEs cells with VNP-shCCDC25 in vitro after obtaining primary mouse NEs and examined the expression levels of N1 and N2 polarization markers of NEs, respectively. qPCR results showed that VNP-shCCDC25 could significantly promote the polarization of NEs toward the N1 antitumor phenotype and significantly inhibit the polarization of NEs toward the N2 protumor phenotype (Fig. 2A and B).

After that, we focused our attention on Mφs. qPCR revealed that VNP-shCCDC25 stimulated the upregulation of *TNF-α*, *IFN-γ*, and *iNOS* (M1-like polarization markers [17, 18]) (Fig. 2C) and downregulated the expression of *FIZZ1* and *Arg-1* (M2-like polarization markers [19, 20]) in RAW264.7 cells (Fig. 2D). These findings indicate that VNP-shCCDC25 can effectively stimulate RAW264.7 cells to polarize toward the antitumor M1-like phenotype and inhibit their polarization toward the protumor M2-like phenotype. These results also suggest that VNP-shCCDC25 could achieve certain antitumor effects by promoting M1-like polarization of Mφs in the TME. Therefore, we simulated this process in vitro by coincubating bacterially stimulated RAW264.7 cell medium with B16F10 cells to determine its effect on the apoptosis of B16F10 cells (Fig. 2E). The results showed that the percentages of apoptotic cells in the VNP-NC and VNP-shCCDC25 groups exceeded 20%, which were significantly greater than that in the control group (Fig. 2F and Additional file 1: Fig. S1A). In addition, direct bacterial stimulation by B16F10 cells competes for nutrients, secretes toxins, and eventually significantly promotes apoptosis, and the apoptosis rate may reach approximately 20% (Fig. 2G and Additional file 1: Fig. S1B). Overall, VNP-shCCDC25 can kill tumor cells directly and stimulate antitumor RAW264.7 cells activation to indirectly kill tumor cells.

After that, B16F10 cells were co-cultured with RFP-expressing VNP (VNP-RFP), and the results showed that VNP-RFP could infect B16F10 cells in vitro (Fig. 2H). Furthermore, after VNP-shCCDC25 was co-incubated with B16F10 cells, we lysed B16F10 cells and counted the titers of bacteria in them. The results showed that the infestation ability of VNP-shCCDC25 was not significantly different from that of VNP-NC (Fig. 2I). These results indicate that VNP-shCCDC25 could infect tumor cells and release shCCDC25 after intracellular cleavage. Furthermore, VNP-shCCDC25 was co-cultured with B16F10 cells, and *CCDC25* mRNA expression was determined by qPCR after successful infection. The results showed that *CCDC25* expression was knocked down to approximately 35% of the original level in B16F10 cells (Fig. 2J).

Taken together, these results confirmed that VNP-shCCDC25 can effectively stimulate RAW264.7 cells activation, infect tumor cells and successfully deliver shCCDC25 in vitro.

### VNP-shCCDC25 inhibits lung metastases from B16F10 cells and orthotopic lung metastases from 4T1 in situ tumors

**Fig. 3:**
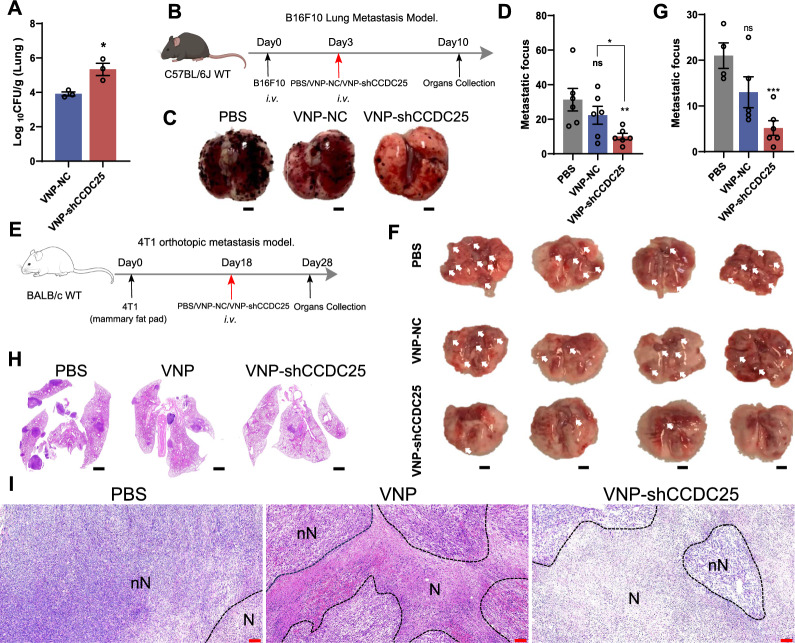
VNP-shCCDC25 inhibits tumor metastasis. **A** The titer of bacterium colonized in the lung tumor at 4 h post *i.v.* with 1 × 10^6^ CFU VNP-NC or VNP-shCCDC25 (*n* = 3). **B** The treatments schedule for VNP-shCCDC25 inhibition of tumor metastasis in B16F10 lung metastasis model: 3 days after B16F10 cells injection, PBS, VNP-NC, or VNP-shCCDC25 was administrated by *i.v.* and the tissues were collected at 10 days. **C**, **D** The pictures and numbers of the lung metastasis foci in B16F10 lung metastasis model (*n* = 6). Scale bars: 2000 μm. **E** The treatment schedule for VNP-shCCDC25 inhibition of tumor metastasis in 4T1 orthotopic lung metastasis model: 18 days after 4T1 cells injection, PBS, VNP-NC, or VNP-shCCDC25 was administrated by *i.v.* and the tissues were collected at 28 days. **F**, **G** The pictures and numbers of the lung metastasis foci in 4T1 orthotopic lung metastasis model. Scale bars: 2000 μm. **H** H&E staining of the lung metastasis foci after treatments in 4T1 orthotopic lung metastasis model. Scale bars: 1000 μm. **I** H&E staining of situ tumors after treatments in 4T1 orthotopic lung metastasis model. Scale bar: 200 µm. N: necrotic region, nN: nonnecrotic region. Data are shown as the mean ± SD. **** p < 0.0001, *** p < 0.001, ** p < 0.01, * p < 0.05, ns: no significance

After establishing that VNP-shCCDC25 has good immunostimulatory and cell-infecting properties, we explored its potential to inhibit tumor metastasis in vivo. Numerous studies have shown that the hypoxic state of the TME is conducive to the proliferation of *Salmonella* [12, 21]. Therefore, we first investigated whether VNP-shCCDC25 could effectively reach tumor sites. In the B16F10 lung metastasis model, after tail vein injection, more VNP-shCCDC25 than VNP-NC cells reached the lung tissue (Fig. 3A), which also provided the foundation for its role in inhibiting tumor metastasis. Afterward, B16F10 lung metastasis model mice were randomly divided into three groups and injected intravenously (*i.v.*) with PBS, VNP-NC, or VNP-shCCDC25 (Fig. 3B). Ultimately, by counting lung metastatic foci, VNP-shCCDC25 was shown to significantly inhibit the lung metastasis of B16F10 cells (Fig. 3C, D).

To further determine the broader application of VNP-shCCDC25 in inhibiting tumor metastasis, and to more realistically mimic the process of cancer metastasis, a 4T1 orthotopic tumor model was generated. Similarly, the model mice were randomly divided into 3 groups and injected *i.v.* with PBS, VNP-NC, or VNP-shCCDC25 (Fig. 3E). Imaging and H&E staining of lung tissue samples showed that VNP-shCCDC25 could significantly inhibit the lung metastasis of in situ 4T1 tumors (Fig. 3F–H). In addition, VNP-shCCDC25 also inhibited the growth of 4T1 in situ tumors to some extent (Additional file 1: Fig. S2A). H&E staining revealed some degree of necrosis of in situ tumors in the VNP-shCCDC25 group (Fig. 3I).

Taken together, these findings demonstrated that VNP-shCCDC25 effectively inhibited metastasis in both of the aforementioned lung metastasis models.

### In vivo safety of VNP-shCCDC25

**Fig. 4:**
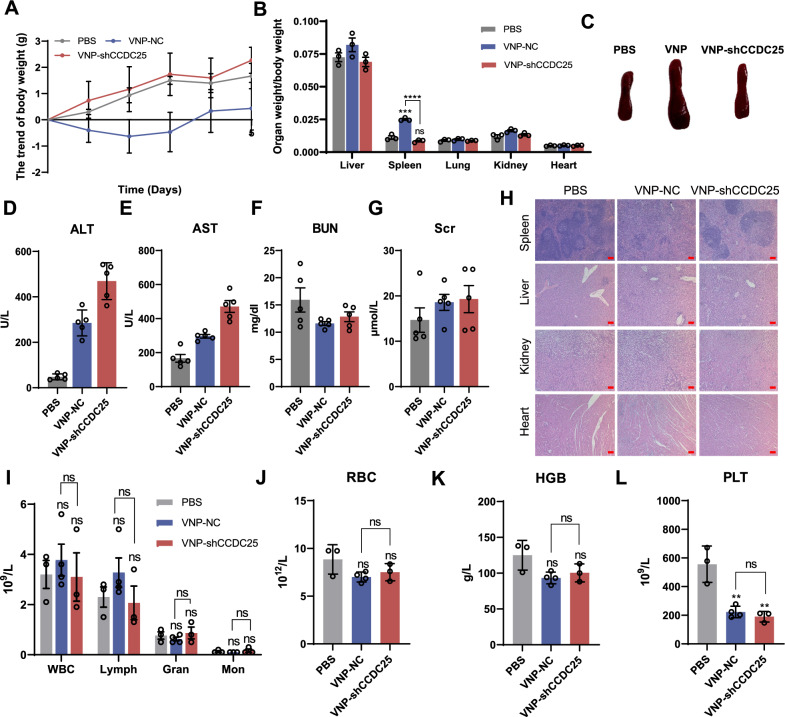
Safety assessment of VNP-shCCDC25 in vivo. **A** The changing trend of body weight of mouse was monitored daily after treatments (*n* = 6). **B** The ratio of organ weight to body weight of mouse in different administration groups (*n* = 3). **C** The picture of spleen after administrations. **D**–**G** Serological analysis of ALT (**D**), AST (**E**), BUN (**F**), and Scr (**G**) in serum 5 days after treatments (*n* = 5). **H** H&E staining of organs in different administration groups. Scale bars: 200 μm. **I**–**L** Routine blood test after 5 days of treatment (*n* = 5). Data are shown as the mean ± SD. **** p < 0.0001, *** p < 0.001, ** p < 0.01, * p < 0.05, ns: no significance

While VNP-shCCDC25 was applied for cancer treatment, the safety of VNP-shCCDC25 was evaluated in vivo. In the B16F10 lung metastasis model, C57BL/6 J mice were randomly divided into 3 groups and injected *i.v.* with PBS, VNP-NC, or VNP-shCCDC25; body weight was monitored daily, and the mice were sacrificed on Day 5. Surprisingly, VNP-shCCDC25 was significantly better than VNP-NC in terms of systemic and organ toxicity. Specifically, body weight, the main indicator of systemic toxicity [22–24], did not decrease after VNP-shCCDC25 administration (Fig. 4A). Additionally, liver and spleen weights are common indicators used to assess the toxicity of related treatments [24], and our results showed that VNP-shCCDC25 did not cause any significant increase in organ weight, especially in the spleen. In contrast, VNP-NC caused significant hepatosplenomegaly (Fig. 4B and C). Serological analysis further revealed that VNP-shCCDC25 caused slight liver damage (Fig. 4D, E) but not significant kidney damage (Fig. 4F, G).

In addition, the H&E staining results also showed that VNP-shCCDC25 did not cause significant organic changes in the organs (Fig. 4H). Considering that VNP-shCCDC25 is a pathogenic microorganism, we also examined the blood routine of mice after administration. The results showed that neither VNP-NC nor VNP-shCCDC25 caused significant hematologic changes except for platelet count (PLT) (Fig. 4I–L). The full names and normal ranges of routine blood test are shown in the Additional file 1: Table S3. It has been shown that the mixed viral-bacterial infection can accompany by a low PLT [25]. Moreover, by monitoring the body weight of 4T1 model mice during treatment and the weight of organs after sacrifice, VNP-shCCDC25 was found to not cause significant systemic toxicity or organ toxicity (Additional file 1: Fig. S2B, C).

Overall, we concluded that VNP-shCCDC25 not only can effectively inhibit cancer metastasis but also has a good biosafety profile.

### VNP-shCCDC25 can inhibit metastasis by blocking the *CCDC25* downstream prometastatic signaling pathway and reducing the formation of NETs

**Fig. 5:**
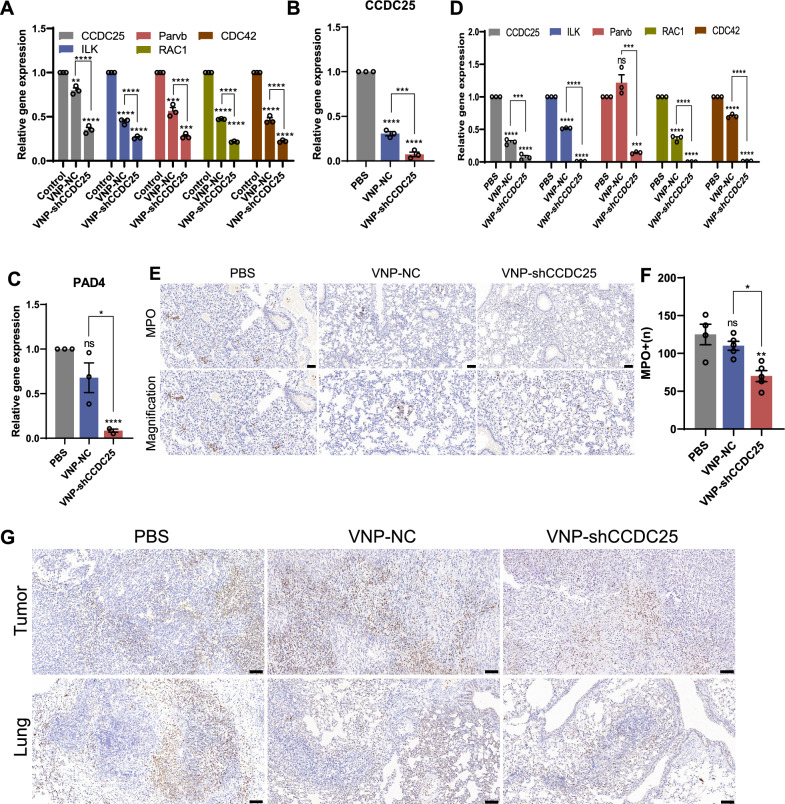
VNP-shCCDC25 blocks the downstream pro-metastasis signaling pathway of *CCDC25* and reduces the formation of NETs. **A** 16 h after co-incubation with VNP-shCCDC25 and B16F10 cells (MOI = 10:1), the expression level of genes in the downstream prometastasis signaling pathway of *CCDC25* in B16F10 cells was analyzed via qPCR. **B** After VNP-shCCDC25 treatment, the expression level of *CCDC25* in the metastatic foci of B16F10 lung metastasis model was analyzed via qPCR. **C** After VNP-shCCDC25 treatment, the expression level of *PAD4* in the metastatic foci of B16F10 lung metastasis model was analyzed via qPCR. **D** In 4T1 orthotopic lung metastasis model, the expression level of genes in the downstream prometastasis signaling pathway of *CCDC25* in the lung tissue after administrations was analyzed via qPCR. **E**, **F** The number of MPO in metastatic foci of B16F10 lung metastasis model was analyzed via immunohistochemistry (IHC). **G** IHC analysis of MPO expression in metastatic foci of 4T1 orthotopic lung metastasis model. Data are shown as the mean ± SD. **** p < 0.0001, *** p < 0.001, ** p < 0.01, * p < 0.05, ns: no significance

In recent years, an increasing number of studies have identified NEs as accomplices in tumor development and metastasis [26, 27], and the mechanisms involved include the secretion of NETs by NEs to mediate tumor metastasis [2, 28]. Specifically, NETs-DNA first binds to CCDC25 on the surface of tumor cells, which in turn enhances the invasiveness of tumor cells by activating the prometastatic *ILK-Parvb-RAC1-CDC42* signaling cascade [7]. Therefore, the expression levels of *CCDC25*-related downstream genes were evaluated after knocking down *CCDC25 *in vitro. VNP-shCCDC25 was co-incubated with B16F10 cells, VNP-shCCDC25 was used to infect B16F10 cells, and *CCDC25* was knocked down. qPCR was subsequently performed to detect the expression levels of the *ILK*, *Parvb*, *RAC1*, and *CDC42* genes. The results showed that all four related genes were significantly downregulated after knocking down *CCDC25* (Fig. 5A). In the TME, the production of NETs is associated with not only NEs but also tumor cells, which can induce the formation of NETs and thus support tumor progression and metastasis [7, 29, 30]. In the present study, *CCDC25* was successfully downregulated at metastatic sites and significantly attenuated cancer metastasis. Therefore, it is reasonable to speculate that the expression or activity of prometastatic genes downstream of *CCDC25* in tumor cells and the formation of NETs at tumor sites would also be affected. Thus, *CCDC25* was analyzed at metastatic sites in the B16F10 lung metastasis model, and the results indicated that VNP-shCCDC25 could successfully knock down *CCDC25* (Fig. 5B).

In addition, peptidyl arginine deiminase 4 (PAD4) is a key substance in the formation of NETs. The expression level of the *PAD4* gene was significantly downregulated in the VNP-shCCDC25 group at lung metastasis foci (Fig. 5C). Similarly, the mRNA expression levels of the downstream prometastatic signaling pathway of *CCDC25* in the 4T1 orthotopic lung metastasis model were analyzed via qPCR, and the results revealed that the gene expression patterns were consistent with the in vitro trends (Fig. 5D).

The levels of NETs in lung tissues were measured by immunohistochemistry in the B16F10 lung metastasis model, and the results showed that VNP-shCCDC25 significantly reduced the levels of NETs in the metastases (Fig. 5E, F). Furthermore, in the PBS and VNP-NC groups, MPO exhibited a diffuse distribution with a punctate pattern; in contrast, the distribution of MPO in the VNP-shCCDC25 group was dense and approximated the cell contours. These results suggested that VNP-shCCDC25 could reduce the formation of NETs. In the 4T1 orthotopic lung metastasis model, the numbers of MPO-positive cells significantly decreased in the VNP-shCCDC25 group and increased in the VNP-NC group (Fig. 5G). The same trends were observed in both orthotopic tumor and lung metastasis foci.

These results suggest that the downregulation of *CCDC25* in tumor tissues suppresses the expression of the downstream prometastatic signaling pathway to some extent, while the reduction in overall *PAD4* expression can lead to a decrease in NETs formation, which further explains the reductions in B16F10 and 4T1 lung metastases in the VNP-shCCDC25 group.

### VNP-shCCDC25 promotes antitumor polarization of tumor-infiltrating neutrophils and macrophages

Tumor-infiltrating immune cells are important indicators of cancer prognosis and treatment responsiveness [31].Thus, we next analyzed the infiltration of immune cells in metastatic tissue to further reveal the mechanism by which VNP-shCCDC25 inhibits tumor metastasis.

**Fig. 6:**
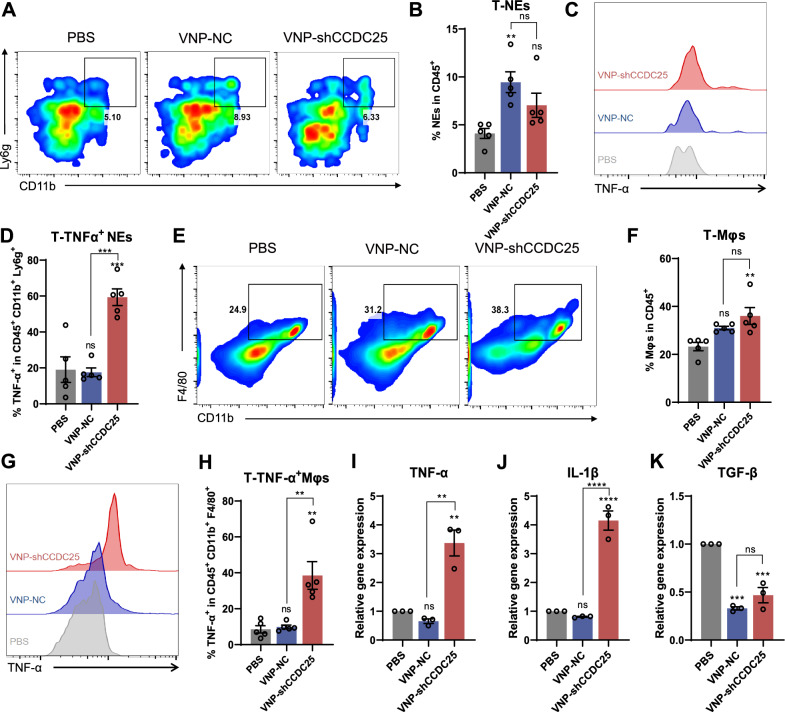
The TME were remodeled after VNP-shCCDC25 administrations. **A**, **B** The percentage of tumor-infiltrating NEs was analyzed via FACS, 5 days after treatments. **C**, **D** The percentage of TNF-α positive cells in tumor-infiltrating NEs was analyzed via FACS. **E** Similarly, the percentage of tumor-infiltrating Mφs was analyzed via FACS, and the statistic diagrams were shown in (**F**). **G** The FACS histogram plot of TNF-α positive cells in tumor-infiltrating Mφs. **H** The statistic diagrams of TNF-α^+^ Mφs in tumor-infiltrating Mφs. **I**-**K** The mRNA expression level of *TNF-α*、*IL-1β*、*TGF-β* in the metastatic foci of B16F10 lung metastasis model was analyzed via qPCR. Data are shown as the mean ± SD. **** p < 0.0001, *** p < 0.001, ** p < 0.01, * p < 0.05, ns: no significance

The percentages of NEs infiltrating the metastases of the PBS, VNP-NC, and VNP-shCCDC25 groups were 4.1%, 9.5%, and 7%, respectively (Fig. 6A, B). Although both VNP-NC and VNP-shCCDC25 recruited more NEs to some extent, only VNP-NC-treated cells showed a significant increase in the infiltration rate of NEs, which was more than two times that of the PBS group. The infiltration of NEs at tumor sites is considered to be a key mediator of tumor transformation, progression, angiogenesis, and regulation of the immune response [32]; however, this does not mean that a higher NE infiltration rate promotes greater tumor progression. This is corroborated by the results of the present study; *i.e.*, the two treatment groups could recruit more NEs to metastases than the PBS group, yet still showed some effect on metastasis inhibition. Therefore, the status of NEs needs to be further investigated. External stimulation of the TME can trigger the switch between the antitumor and protumor phenotypes of TANs that accumulate in local areas [33]. It has been proposed that in the TME, TANs exhibit an N1 phenotype, which is an antitumor phenotype, and an N2 phenotype, which is a protumor phenotype [15]. NEs exert antitumor or protumor effects at tumor sites related to the infiltration rate of their N1- and N2-like phenotypes. In addition, some studies have suggested that N1-related genes include *CCL3*, *ICAM1*, *iNOS*, and *TNF-α* [16]. By combining these results and the results of other studies, we further analyzed the percentage of NEs with a TNF-α^+^NE phenotype (*i.e.*, an N1-like antitumor phenotype) among the NEs among the metastases. As shown in Fig. 6C, D, approximately 59.3% of the NEs recruited by VNP-shCCDC25 were TNF-α^+^ NEs, which was much greater than the 19% of PBS-treated cells and 17.6% of VNP-NC-treated cells and more than three times as many as the other two groups. Specifically, compared to the VNP-NC group, although the infiltration rate of NEs into metastases was slightly lower after VNP-shCCDC25 treatment, the infiltration rate of N1-like antitumor phenotype NEs was significantly greater.

In addition, the infiltration rates of Mφs into metastases in the PBS, VNP-NC, and VNP-shCCDC25 groups were 23.2%, 31%, and 36%, respectively, *i.e.*, the VNP-shCCDC25 group showed a significant increase compared with PBS group, which was approximately 1.6 times higher (Fig. 6E, F). Similarly, as shown in Fig. 6G, H, approximately 38.5% of the Mφs recruited by VNP-shCCDC25 were TNF-α^+^ Mφs (M1 antitumor phenotype Mφs), which was much greater than the 8.5% in PBS and 9.8% in VNP-NC and was approximately fourfold greater than the other two groups. That is, compared with VNP-NC, VNP-shCCDC25 not only recruited more Mφs but also significantly enhanced the infiltration rate of M1-like antitumor Mφs into metastases. Then, we detected the N1/N2 and M1/M2 markers *TNF-α* (N1, M1 marker) [34, 35], *IL-1β* (N1, M1 marker) [36, 37], and *TGF-β* (M2 marker) [38, 39] by qPCR to further confirm the TME after VNP-shCCDC25 treatment. The results showed that *TNF-α* and *IL-1β* expression levels were significantly greater and that *TGF-β* expression was significantly lower in the metastases of the VNP-shCCDC25 group than in those of the control group (Fig. 6I–K). That is, compared with PBS, VNP-shCCDC25 significantly increased the infiltration of antitumor phenotypic immune cells into the metastases. Furthermore, there is evidence that the immunosuppressive cytokine TFG-β is overexpressed in tumors and plays an important role in blocking the immune response and promoting tumor progression and that inhibition of the *TGF-β* signaling pathway increases the infiltration of NEs into tumors [15]. This finding is also consistent with the results of the present study.

Furthermore, we examined the infiltration of Mφs into the spleens, TdLNs, and peripheral blood of mice, which were identified as tumor-related immune organs. The results showed that the infiltration rate of TNF-α^+^ Mφs in the spleens of the VNP-shCCDC25 group was approximately 9.2%, which was significantly greater than the rates in the PBS (1.1%) and VNP-NC (2.1%) groups (Additional file 1: Fig. S3A, B). CD86 is a marker of M1-like Mφs [40], We found that the infiltration rate of CD86^+^ Mφs in TdLNs in the VNP-shCCDC25 group was approximately 18.9%, which was also significantly greater than the rates in the PBS (12.1%) and VNP-NC (15.3%) groups (Additional file 1: Fig. S3C, D). The percentage of CD86^+^ Mφs in the peripheral blood of the VNP-shCCDC25 group was approximately 9.9%, which was more than twofold greater than that in the peripheral blood (4.9% in the PBS group and 3.3% in the VNP-NC group) (Additional file 1: Fig. S3E, F). These results suggest that VNP-shCCDC25 treatment also promotes the polarization of peripheral Mφs toward the M1-like antitumor phenotype, suggesting that VNP-shCCDC25 can remodel the TME.

Overall, we conclude that the levels of M1-like Mφs and N1-like NEs in metastatic tissue as well as in peripheral tumor-related immune organs are significantly increased after VNP-shCCDC25 treatment, and the presence of these antitumor immune cells indirectly explains the inhibition of metastasis by VNP-shCCDC25.

### VNP-shCCDC25 activates antigen presentation by dendritic cells and thus activates antitumor immune response

**Fig. 7:**
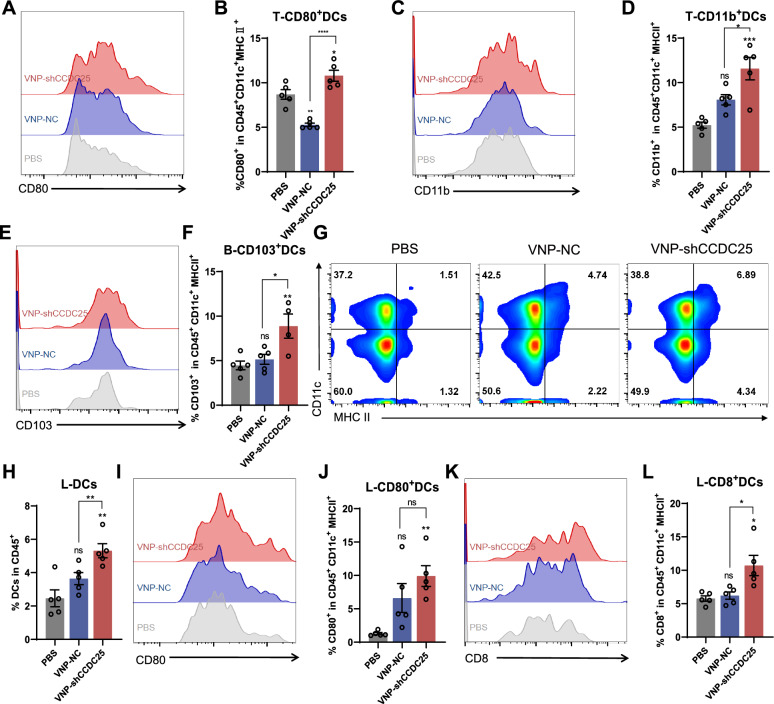
VNP-shCCDC25 stimulated DCs maturation. **A**, **B** The percentage of CD80 positive cells in tumor-infiltrating DCs was analyzed via FACS. **C**, **D** The percentage of CD11b positive cells in tumor-infiltrating DCs was analyzed via FACS. **E**, **F** 5 days after administrations, the level of CD103 in DCs in peripheral blood was analyzed via FACS. **G** The percentage of DCs in TdLNs. And the statistic diagrams were shown in (**H**). **I**, **J** In TdLNs, the percentage of CD80^+^DCs was analyzed via FACS. **K**, **L** Similarly, CD8^+^ DCs in TdLNs was evaluated. Data are shown as the mean ± SD. **** p < 0.0001, *** p < 0.001, ** p < 0.01, * p < 0.05, ns: no significance

Although dendritic cells (DCs) are a rare population of immune cells in tumors and lymphoid organs, they have great potential to induce effective antitumor immunity [31, 41]. Therefore, it is essential to analyze the infiltration of DCs and their subtypes in metastatic foci as well as in the periphery. We found that the infiltration rates of DCs in the metastatic foci of the PBS, VNP-NC, and VNP-shCCDC25 groups were 6.5%, 6.9%, and 6.9%, respectively, and that the infiltration rates of DCs did not change significantly among the groups (Additional file 1: Fig. S4A, B). CD80 is a marker of DC activation [42], and further analysis revealed that the infiltration rate of CD80^+^ DCs in VNP-shCCDC25-treated mouse metastases was 10.8%, which was significantly greater than the infiltration rates in PBS-treated (8.7%) and VNP-NC-treated (5.3%) cells (Fig. 7A, B). These findings indicate that VNP-shCCDC25 can effectively activate metastatic focal DCs.

In addition, mouse conventional DCs (cDCs) include two major subpopulations, namely, the CD8^+^ and/or CD103^+^ subpopulation of cDC1s and the CD11b^+^ subpopulation of cDC2s [31, 43, 44]. CD8^+^ T cells are associated with DC resident cells, whereas CD103^+^ T cells are associated with tumor migration [31]. cDC1s play a key role in antitumor immunity, and knockout mice lacking cDC1s fail to generate antitumor CD8^+^ T-cell responses [45–47]. Moreover, cDC2s are potent inducers of CD4^+^ T-cell responses [48, 49]. Therefore, the expression levels of these markers (CD8, CD103, and CD11b) were analyzed via FACS. The results showed that the infiltration rate of CD11b^+^ DCs into VNP-shCCDC25-treated metastases was 11.6%, which was significantly greater than the infiltration rates of 5.2% in the PBS group and 8.1% in the VNP-NC group (Fig. 7C, D). It indicates that VNP-shCCDC25 could effectively activate T-cell immunity in metastases. The percentage of CD103^+^ DCs in the peripheral blood was 8.9% in the VNP-shCCDC25 group, which was significantly greater than the percentages of 4.5% in the PBS group and the 5.1% in the VNP-NC group (Fig. 7E, F). It is implied that DCs in peripheral blood can migrate to tumor-associated immune organs to activate antitumor immunity. The infiltration rate of DCs in TdLNs increased to 5.3% in the VNP-shCCDC25 group, which was significantly greater than the infiltration rates in the PBS (2.5%) and VNP-NC (3.6%) groups (Fig. 7G, H). Furthermore, when detecting activated DCs in TdLNs, we found that the infiltration rate of CD80+ DCs in the VNP-shCCDC25 group was 9.9%, which was significantly greater than the infiltration rates in the PBS (1.3%) and VNP-NC (6.6%) groups (Fig. 7I, J). These findings suggested that VNP-shCCDC25 can effectively activate DCs in TdLNs. After that, we examined CD8^+^ DCs in TdLNs and found that the infiltration rate in the VNP-shCCDC25 group was 10.7%, which was significantly greater than the infiltration rates in the PBS (5.8%) and VNP-NC (6.2%) groups (Fig. 7K, L). These findings indicate that VNP-shCCDC25 can effectively induce antitumor immunity in TdLNs, which in turn inhibits metastasis.

As mentioned previously, cDC1s and cDC2s are associated with CD8^+^ and CD4^+^ T-cell immunity, respectively. Therefore, the statuses of CD8^+^ and CD4^+^ T cells in metastatic foci and TdLNs were evaluated. There was a slight but not significant increase in CD8^+^ T cells in VNP-shCCDC25-treated metastases and a significant decrease in CD4^+^ T cells (Additional file 1: Fig. S4C–E). Among the TdLNs, 5.4% of the PD1^+^ CD8^+^ T cells in the VNP-shCCDC25 group were infiltrated, which was significantly lower than the 18.6% in the PBS group and the 13.9% in the VNP-NC group (Additional file 1: Fig. S4F, G). The infiltration rate of VNP-shCCDC25 TdLNs by CD4^+^PD1^+^ T cells was 4.4%, which was significantly lower than the infiltration rates of 9.6% in the PBS group and 8.3% in the VNP-NC group (Additional file 1: Fig. S4H, I). These results suggest that there is some degree of relief of tumor immunosuppression after VNP-shCCDC25 treatment. Taken together, the above results suggest that VNP-shCCDC25 can activate DCs, in turn activating antitumor immunity and alleviating the state of tumor immunosuppression, ultimately suppressing metastasis.

**Fig. 8:**
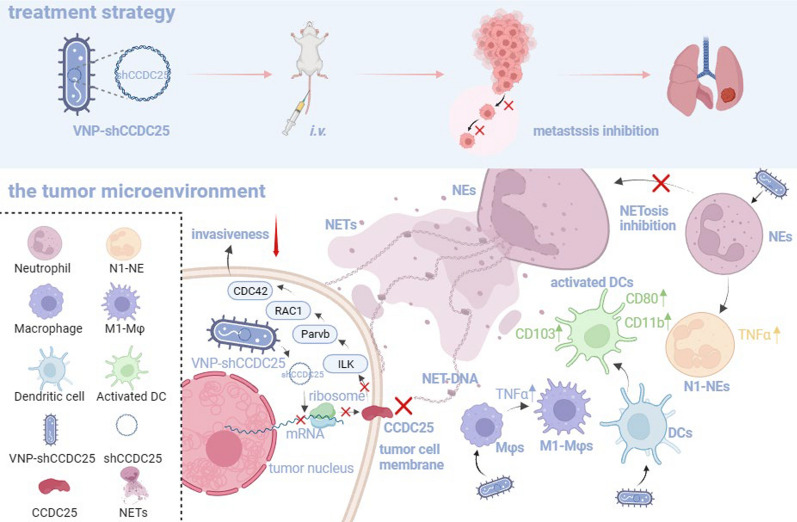
Schematic diagram of VNP-shCCDC25 blocking CCDC25-NET-DNA and remodeling immunity in B16F10-bearing mice and 4T1-bearing mice. Overall, VNP-shCCDC25 inhibits tumor lung metastasis after intravenous injection in mice. Specifically, VNP-shCCDC25 mediates tumor lung metastasis inhibition through the following 3 mechanisms. (1) VNP-shCCDC25 knocked down *CCDC25* and its downstream prometastasis signaling pathway *ILK-Parvb-RAC1-CDC42* after successful invasion of tumor cells, which in turn reduced the invasiveness of tumor cells. (2) The NETosis at tumor site was somewhat inhibited after knocking down *CCDC25*. (3) In TME, elevated infiltration rates of M1-Mφs and N1-NEs with antitumor phenotypes as well as effective activation of DCs were observed, suggesting that VNP-shCCDC25 can remodel TME and ultimately achieve tumor metastasis suppression

Overall, VNP-shCCDC25 effectively knocked down *CCDC25* and its downstream prometastatic signaling pathway after the successful invasion of tumor cells, which in turn reduced their invasiveness. In addition, the formation of NETs at the tumor site was somewhat inhibited after knocking down *CCDC25*, which explains the inhibition of tumor metastasis. More surprisingly, elevated infiltration of Mφs and NEs with antitumor phenotypes and effective activation of DCs were observed at tumor sites and even in peripheral tumor-associated immune organs, suggesting that the tumor model mice exhibited good responsiveness to VNP-shCCDC25 treatment, which lays a solid foundation for the effective inhibitory effect of VNP-shCCDC25 on metastasis (Fig. 8).

## Discussion

A large amount of evidence indicates the presence of NETs in peripheral blood and tumor specimens from animal models and cancer patients, which can capture cancer cells and act as adhesion substrates, thus promoting cancer metastasis [4, 50–52]. Therefore, reducing the formation of abnormal NETs or enhancing the degradation of NETs is a therapeutic strategy for inhibiting cancer metastasis. Previous works have focused on PAD4, a key substance involved in NET formation [3]. The PAD4 inhibitor C1-amidine was found to reduce the formation of NETs, but its serum half-life ranges from only 15 min to 4 h, and it can interact with other PAD family enzymes and lacks specificity, thus limiting its clinical application [53, 54]. Granulocyte colony-stimulating factor (G-CSF) is commonly used in the clinic to prevent deaths associated with reduced NEs, and 4T1 cells can secrete G-CSF to induce NEs to form NETs. Additionally, anti-G-CSF can reduce the ability of 4T1 cells to induce NETs, but significant side effects can occur with treatment [53, 55, 56]. After pathogen induction, NEs are activated, and NETs are released; the first step in this signaling cascade is the activation of the NADPH oxidase complex (NOX2) [53, 57]. As mentioned previously, NETs are formed when DNA is modified by substances such as neutrophil elastase (NE), i.e., NE is required for NETs release [58, 59]. Both the NOX2 inhibitor apocynin and the NE inhibitor sivelestat reduce the ability of NEs to form NETs [53]. However, NOX2 and NE are also not good targets, as both are essential for the killing of pathogenic bacteria by NEs. In addition, some studies have shown that DNase I degrades NETs, leading to loss of reticulation and a reduced ability to promote metastasis [60, 61], however, its serum half-life is short, and its effect is relatively limited [53, 62]. Overall, we see that different anti-NET treatments face limitations, and there is an urgent need to explore new approaches of anti-NETs.

The DNA component of NETs has a chemotactic effect on tumor cells by interacting with CCDC25 on the surface of tumor cells with high affinity, and DNA-CCDC25 interactions trigger intracellular signaling cascade responses that ultimately promote the migration and metastasis of tumor cells [7]. In mouse models, treatment with anti-CCDC25 neutralizing antibodies reduced NET-mediated metastasis, suggesting that targeting CCDC25 may be a novel therapeutic strategy for metastasis prevention [3]. However, antibody drugs are associated with high production costs and the need for multiple doses. In contrast, nucleic acid drugs are simple to prepare, significantly less expensive to produce, and require only a single administration, but the key limitation is the difficulty of in vivo delivery. VNP was shown to be safe in phase I clinical trials, and numerous studies have demonstrated that VNP is a suitable vehicle for nucleic acid delivery [9, 24]. Furthermore, VNP has been used as a potent antitumor agent for targeted therapy due to its selective accumulation in tumors [63]. Therefore, in the present study, *CCDC25* was successfully knocked down in tumor tissues by safely delivering shCCDC25 to tumor sites via VNP, which ultimately significantly inhibited tumor metastasis and demonstrated high efficiency and low toxicity in several tumor models. The tumor growth inhibition rates reached 68.082% and 83.509% in the B16F10 lung metastasis and 4T1 orthotopic lung metastasis models, respectively (Additional file 1: Table S4).

Furthermore, we found that the TME was also altered after anti-CCDC25 therapy; for example, the infiltration rates of M1-like Mφs and N1-like NEs with antitumor phenotypes were significantly increased, and DCs were also sufficiently activated to trigger antitumor immunity; more importantly, the level of NETs was also significantly reduced. These results also indirectly reveal the mechanism by which anti-CCDC25 treatment inhibits tumor metastasis. Overall, our study explored a novel anti-NET strategy for the first time by using an oncolytic bacteria-mediated delivery system in which the CCDC25 nucleic acid drug was used to target *CCDC25*, a key gene for tumor cell metastasis, and showed promising application in terms of both safety and the inhibition of tumor metastasis.

## Conclusion

In this study, we effectively knocked down *CCDC25* by delivering nucleic acid drugs to tumor sites through VNP, a microbial delivery system, and ultimately successfully inhibited tumor metastasis in several tumor models. This study explored a new anti-NETs strategy, and the first proposed anti-CCDC25 therapy showed promise for inhibiting cancer metastasis.

### Supplementary Information

Additional file 1: **Figure S1.** **A** The apoptosis levels of B16F10 cells after incubated for 16 hours with RAW264.7 cell medium, which was stimulated with VNP-NC, VNP-shCCDC25, or not (*n*=3). **B** The apoptosis levels of B16F10 cells after incubated with VNP-NC,VNP-shCCDC25, or not for 16 hours (*n*=3). **Figure S2.** **A** The trend of tumor volume of *in-situ* tumors in 4T1 orthotopic lung metastasis model (*n*=6). **B** The changing trend of body weight of BALB/c was monitored daily after *i.v.* of PBS, VNP-NC or VNP-shCCDC25. **C** The ratio of organ weight to body weight of of BALB/c in different administration groups after sacrifice. Data are shown as the mean ± SD. **** p < 0.0001, *** p < 0.001, ** p < 0.01, * p < 0.05, ns: no significance. **Figure S3.** **A** The FACS histogram plot of TNF-α^+^ Mφs in spleen. **B** The statistic diagram of TNF-α^+^ Mφs in spleen. **C, D** The percentage of CD86 positive cells in Mφs in TdLNs was analyzed via FACS. **E, F** Similarly, the percentage of CD86^+^ Mφs in peripheral blood after administrations was analyzed via FACS. Data are shown as the mean ± SD. **** p < 0.0001, *** p < 0.001, ** p < 0.01, * p < 0.05, ns: no significance. **Figure S4.** **A, B** The percentage of tumor-infiltrating DCs was analyzed via FACS. **C–E** The percentage of tumor-infiltrating CD8^+^ T cells (**D**) and CD4^+^ T cell (**E**). **F, G** The percentage of PD1^+^CD8^+^ T cells in TdLNs. **H, I** Similarly, the percentage of PD1^+^CD4^+^ T cells in TdLNs 5 days after administrations was analyzed via FACS. Data are shown as the mean ± SD. **** p < 0.0001, *** p < 0.001, ** p < 0.01, * p < 0.05, ns: no significance. **Table S1.** The primer sequence of RT-PCR. **Table S2.** The information of FACs antibody. **Table S3.** Full names and normal ranges of routine blood test. **Table S4.** The tumor inhibition efficacy of VNP-shCCDC25.

## Data Availability

Without restrictions.
