# Supplementary material for: The oncolytic bacteria-mediated delivery system of CCDC25 nucleic acid drug inhibits neutrophil extracellular traps induced tumor metastasis
Source: J Nanobiotechnology. 2024 Feb 19;22:69. doi: 10.1186/s12951-024-02335-5 (PMC10875894; doi:10.1186/s12951-024-02335-5)
Supplement: Supplementary file 1 — Additional file 1: Figure S1. A The apoptosis levels of B16F10 cells after incubated for 16 hours with RAW264.7 cell medium, which was stimulated with VNP-NC, VNP-shCCDC25, or not (n=3). B The apoptosis levels of B16F10 cells after incubated with VNP-NC,VNP-shCCDC25, or not for 16 hours (n=3). Figure S2. A The trend of tumor volume of in-situ tumors in 4T1 orthotopic lung metastasis model (n=6). B The changing trend of body weight of BALB/c was monitored daily after i.v. of PBS, VNP-NC or VNP-shCCDC25. C The ratio of organ weight to body weight of of BALB/c in different administration groups after sacrifice. Data are shown as the mean ± SD. **** p < 0.0001, *** p < 0.001, ** p < 0.01, * p < 0.05, ns: no significance. Figure S3. A The FACS histogram plot of TNF-α+ Mφs in spleen. B The statistic diagram of TNF-α+ Mφs in spleen. C, D The percentage of CD86 positive cells in Mφs in TdLNs was analyzed via FACS. E, F Similarly, the percentage of CD86+ Mφs in peripheral blood after administrations was analyzed via FACS. Data are shown as the mean ± SD. **** p < 0.0001, *** p < 0.001, ** p < 0.01, * p < 0.05, ns: no significance. Figure S4. A, B The percentage of tumor-infiltrating DCs was analyzed via FACS. C–E The percentage of tumor-infiltrating CD8+ T cells (D) and CD4+ T cell (E). F, G The percentage of PD1+CD8+ T cells in TdLNs. H, I Similarly, the percentage of PD1+CD4+ T cells in TdLNs 5 days after administrations was analyzed via FACS. Data are shown as the mean ± SD. **** p < 0.0001, *** p < 0.001, ** p < 0.01, * p < 0.05, ns: no significance. Table S1. The primer sequence of RT-PCR. Table S2. The information of FACs antibody. Table S3. Full names and normal ranges of routine blood test. Table S4. The tumor inhibition efficacy of VNP-shCCDC25. [file 12951_2024_2335_MOESM1_ESM.docx]

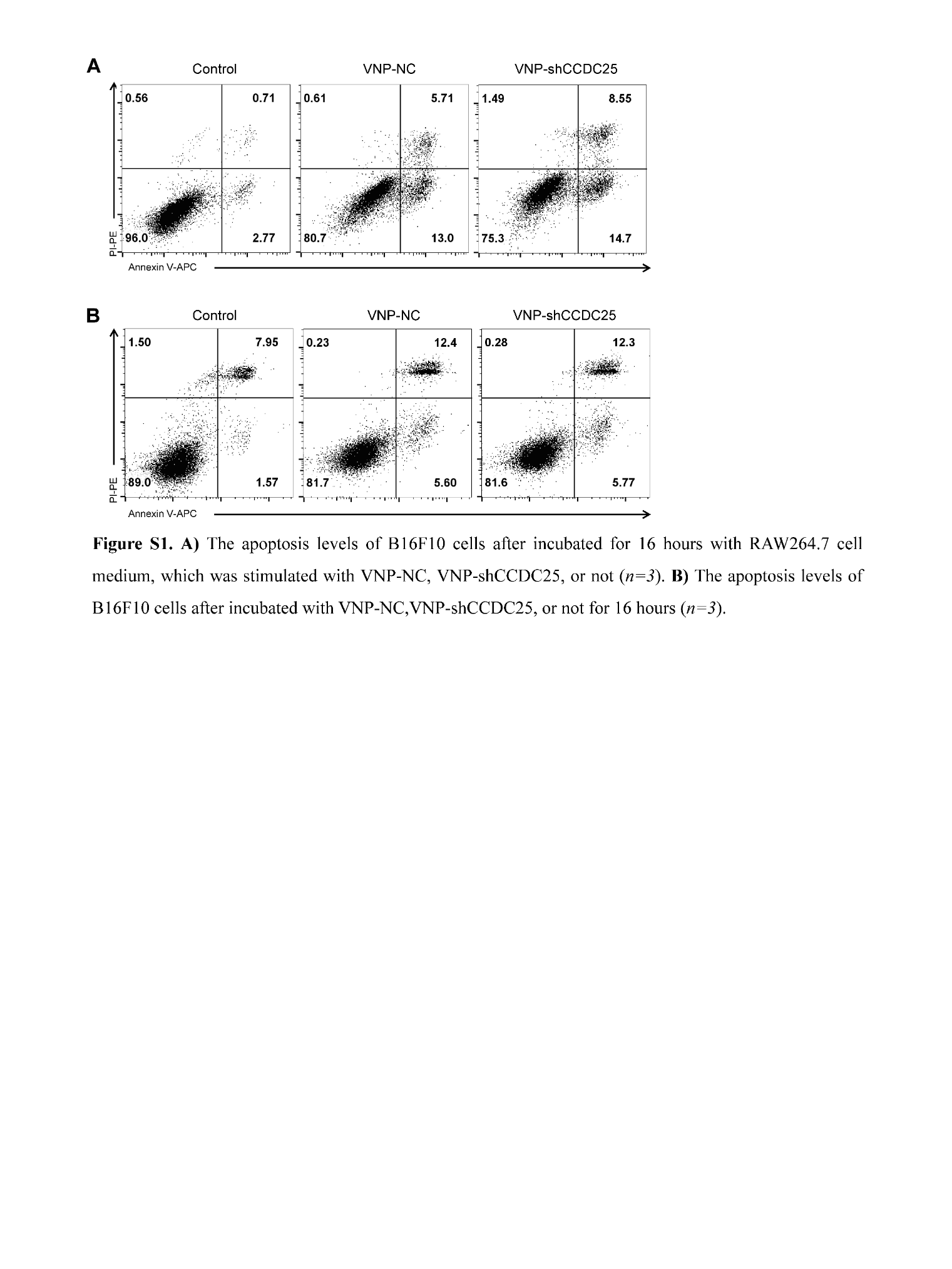


**Fig. S1. A)** The apoptosis levels of B16F10 cells after incubated for 16 hours with RAW264.7 cell medium, which was stimulated with VNP-NC, VNP-shCCDC25, or not (*n*=3). **B)** The apoptosis levels of B16F10 cells after incubated with VNP-NC,VNP-shCCDC25, or not for 16 hours (*n*=3).


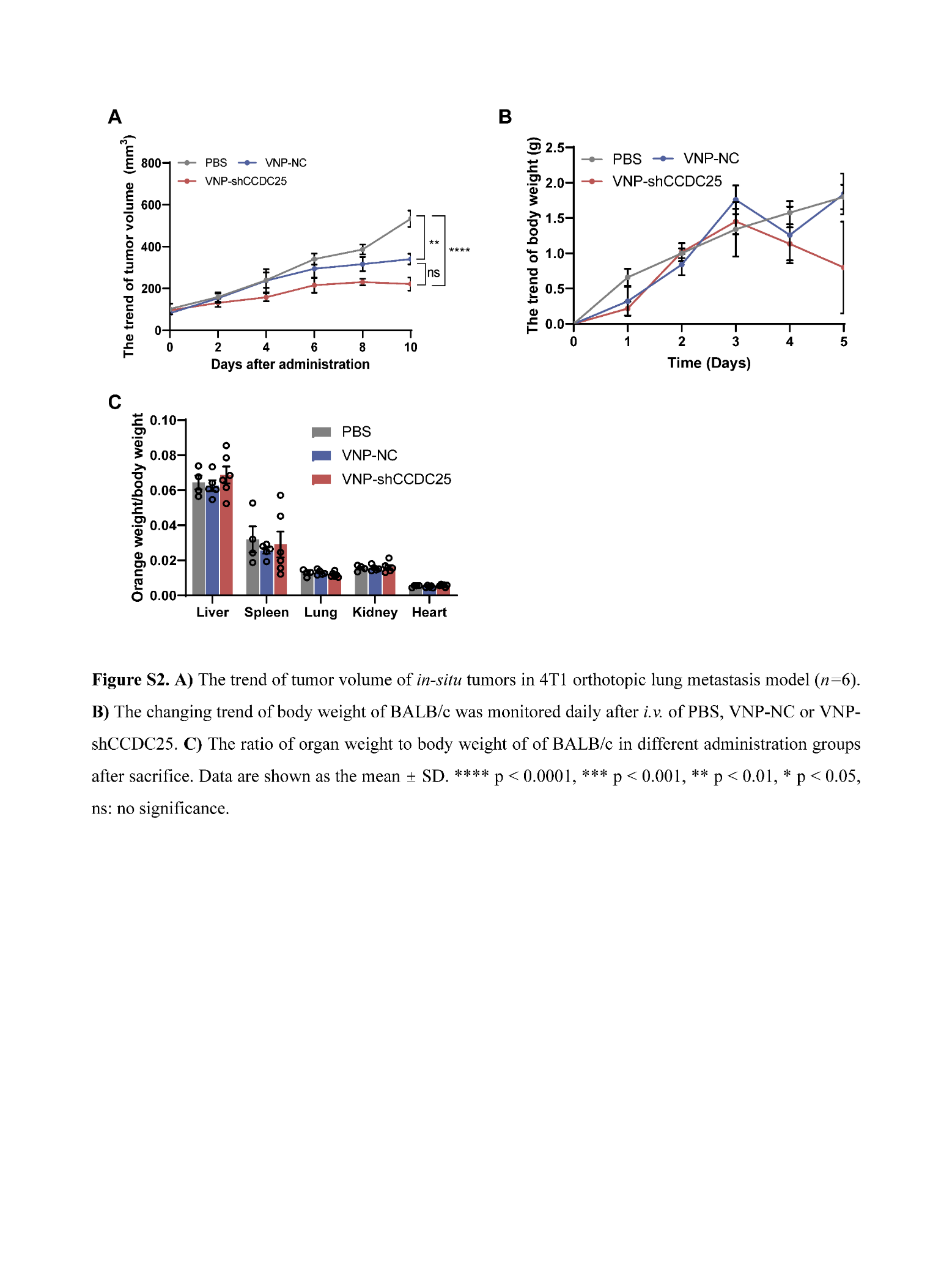


**Fig. S2. A)** The trend of tumor volume of *in-situ* tumors in 4T1 orthotopic lung metastasis model (*n*=6). **B)** The changing trend of body weight of BALB/c was monitored daily after *i.v.* of PBS, VNP-NC or VNP-shCCDC25. **C)** The ratio of organ weight to body weight of of BALB/c in different administration groups after sacrifice. Data are shown as the mean ± SD. **** p < 0.0001, *** p < 0.001, ** p < 0.01, * p < 0.05, ns: no significance.


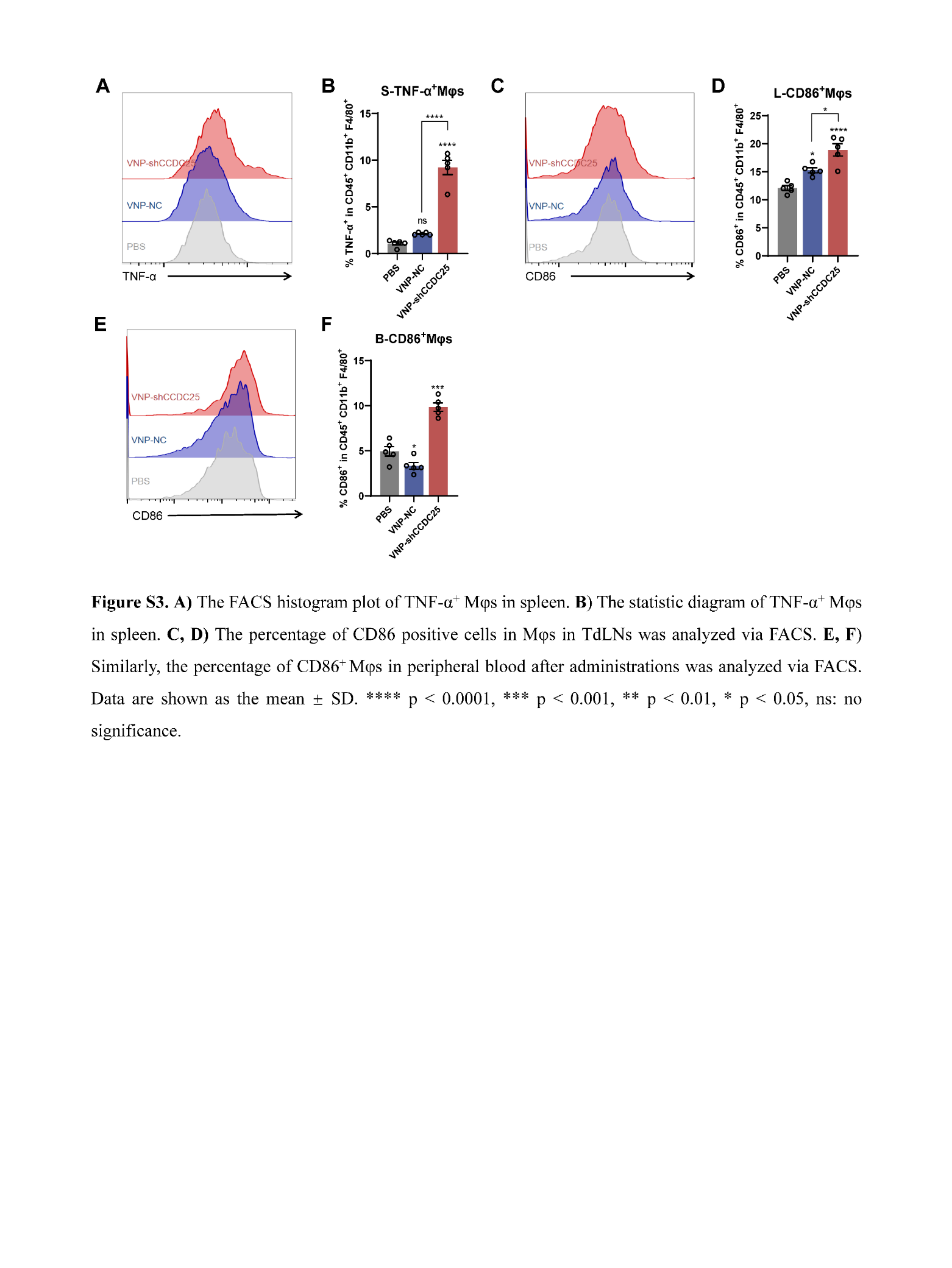


**Fig. S3. A)** The FACS histogram plot of TNF-α^+^ Mφs in spleen. **B)** The statistic diagram of TNF-α^+^ Mφs in spleen. **C, D)** The percentage of CD86 positive cells in Mφs in TdLNs was analyzed via FACS. **E, F)** Similarly, the percentage of CD86^+^ Mφs in peripheral blood after administrations was analyzed via FACS. Data are shown as the mean ± SD. **** p < 0.0001, *** p < 0.001, ** p < 0.01, * p < 0.05, ns: no significance.


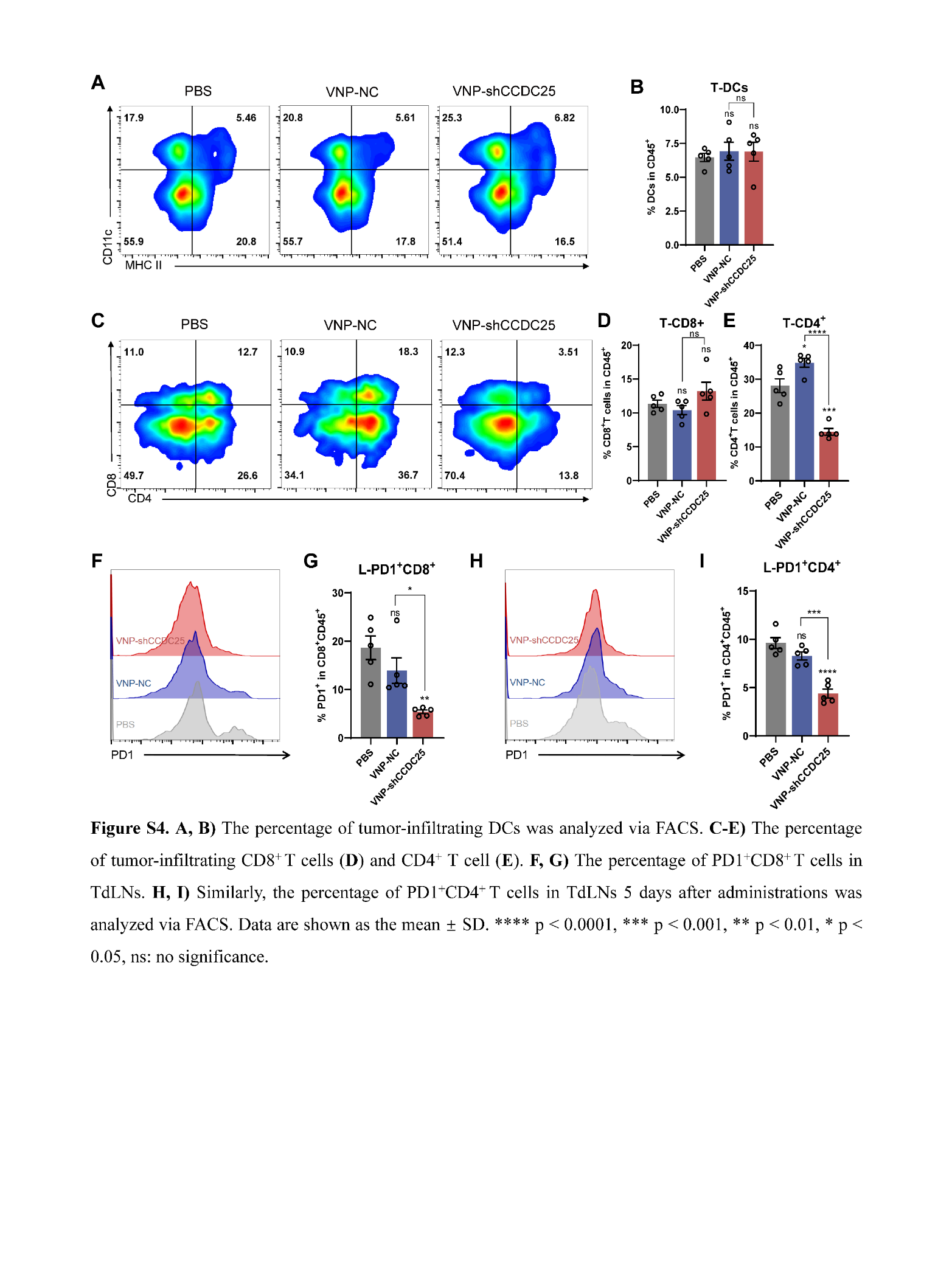


**Fig. S4.** **A, B)** The percentage of tumor-infiltrating DCs was analyzed via FACS. **C-E)** The percentage of tumor-infiltrating CD8^+^ T cells (**D**) and CD4^+^ T cell (**E**). **F, G**) The percentage of PD1^+^CD8^+^ T cells in TdLNs. **H, I)** Similarly, the percentage of PD1^+^CD4^+^ T cells in TdLNs 5 days after administrations was analyzed via FACS. Data are shown as the mean ± SD. **** p < 0.0001, *** p < 0.001, ** p < 0.01, * p < 0.05, ns: no significance.

**Table S1. The primer sequence of RT-PCR**

| **Primer** | **Forward (5'-3')** | **Reverse（5'-3'）** |
| --- | --- | --- |
| 18s | GTAACCCGTTGAACCCCATT | CCATCCAATCGGTAGTAGCG |
| IFN-γ | GCCACGGCACAGTCATTGA | TGCTGATGGCCTGATTGTCTT |
| TNF-α | ACCACGCTCTTCTGTCTACT | AGGAGGTTGACTTTCTCCTG |
| iNos | GTTCTCAGCCCAACAATACAAGA | GTGGACGGGTCGATGTCAC |
| Arg-1 | GATTGGCAAGGTGATGGAAG | TCAGTCCCTGGCTTATGGTT |
| IL-1β | GAAATGCCACCTTTTGACAGTG | TGGATGCTCTCATCAGGACAG |
| Fizz1 | CCAATCCAGCTAACTATCCCTCC | CCAGTCAACGAGTAAGCACAG |
| TGF-β | CTCCCGTGGCTTCTAGTGC | GCCTTAGTTTGGACAGGATCTG |
| CCL3 | ACCATGACACTCTGCAACCA | TCAGGCATTCAGTTCCAGGT |
| CCL2 | CAGGTCCCTGTCATGCTTCT | GTCAGCACAGACCTCTCTCT |
| CCDC25 | TCTTCGGCCCATGTGTACCTA | GGCACAGTCCATCAAAACCTC |
| PAD4 | TCTGCTCCTAAGGGCTACACA | GTCCAGAGGCCATTTGGAGG |
| ILK | ACGACATTTTCACTCAGTGCC | TCATCCCCACGATTCATCACAT |
| Parvb | AAGGACGAGTCTTTCTTGGGC | GGGGCCATTGGAGAGTTGAT |
| CDC42 | CCCATCGGAATATGTACCAACTG | CCAAGAGTGTATGGCTCTCCAC |
| Rac1 | GAGACGGAGCTGTTGGTAAAA | ATAGGCCCAGATTCACTGGTT |

**Table S2. The information of FACs antibody**

| **Antibodies** | **Article No.** | **Company** |
| --- | --- | --- |
| Ms CD45-PE Cy7 | 552848 | BD Pharmingen™ |
| Ms CD8a-PerCP Cy5.5 | 551162 | BD Pharmingen™ |
| Ms CD8-FITC | 553030 | BD Pharmingen™ |
| Ms CD11c-PE | 553802 | BD Pharmingen™ |
| Ms I-A/I-E BB700 | 746197 | BD Pharmingen™ |
| Ms CD11b- PerCP Cy5.5 | 550993 | BD Pharmingen™ |
| Ms CD11b- APC | 557396 | BD Pharmingen™ |
| Ms CD80-BV421 | 562611 | BD Pharmingen™ |
| Ms CD206-AF647 | 565250 | BD Pharmingen™ |
| Ms CD69-BV421 | 562920 | BD Pharmingen™ |
| Ms F4/80-BV510 | 743280 | BD Pharmingen™ |
| Ms Ly6C-PE | 560592 | BD Pharmingen™ |
| Ms Ly6G-PE.Cy7 | 560601 | BD Pharmingen™ |
| CD4-PE.Cy7 | 12-0043-82 | eBioscience™ |
| Granzyme B-PE | 12-8898-82 | eBioscience™ |
| PD1-APC | 17-9985-82 | eBioscience™ |
| TNFα-BV421 | 506328 | Biolegend™ |
| CD86-FITC | 105005 | Biolegend™ |
| CD103-PE.Cy7 | 121426 | Biolegend™ |

**Table S3. Full names and normal ranges of routine blood test**

| **Abbreviations** | **Full names** | **Normal ranges** | **Units** |
| --- | --- | --- | --- |
| WBC | White blood cell count | 0.8-10.6 | 10^^^9/L |
| Lymph | Lymphocyte count | 0.6-8.9 | 10^^^9/L |
| Gran | Granulocyte | 0.23-3.6 | 10^^^9/L |
| Mon | Monocyte | 0.04-1.4 | 10^^^9/L |
| RBC | Red blood cell | 6.5-11.5 | 10^^^12/L |
| HGB | Hemoglobin | 110-165 | g/L |
| PLT | Platelet count | 400-1600 | 10^^^9/L |

**Table S4. The tumor inhibition efficacy of VNP-shCCDC25**

| Group | B16F10 lung metastasis model | 4T1 orthotopic lung metastasis model |
| --- | --- | --- |
|  | Tumor growth inhibition rates | Tumor metastatic inhibition rates |
| PBS | 0% | 0% |
| VNP-NC | 28.726% | 58.506% |
| VNP-shCCDC25 | 68.082% | 83.509% |
